# Prospective Evaluation of Cardiopulmonary Resuscitation Performed in Dogs and Cats According to the RECOVER Guidelines. Part 1: Prognostic Factors According to Utstein-Style Reporting

**DOI:** 10.3389/fvets.2019.00384

**Published:** 2019-11-07

**Authors:** Sabrina N. Hoehne, Steven E. Epstein, Kate Hopper

**Affiliations:** ^1^William R. Pritchard Veterinary Medical Teaching Hospital, School of Veterinary Medicine, University of California, Davis, Davis, CA, United States; ^2^Department of Veterinary Surgical and Radiological Sciences, School of Veterinary Medicine, University of California, Davis, Davis, CA, United States

**Keywords:** cardiopulmonary resuscitation, guidelines, cardiac arrest, outcome factors, dog, cat

## Abstract

Factors associated with positive cardiopulmonary resuscitation (CPR) outcomes defined according to the veterinary Utstein-style CPR reporting guidelines have not been described since implementation of the Reassessment Campaign on Veterinary Resuscitation (RECOVER) CPR clinical guidelines in 2012. The aims of this study were to assess factors associated with positive CPR outcomes at a U.S. veterinary teaching hospital, to re-evaluate these factors since implementation of the RECOVER guidelines compared to reported factors prior to their publication, and to identify potential additional factors since guideline publication. One-hundred and seventy-two dogs and 47 cats that experienced cardiopulmonary arrest (CPA) and had CPR performed were prospectively included in this observational study. Supervising clinicians were asked to complete a data form on CPR events immediately following completion of CPR efforts. Multivariable logistic regression was used to evaluate the effect of twenty hospital, animal, and arrest variables on the three patient outcomes “any return of spontaneous circulation (ROSC),” “sustained ROSC,” and survival to hospital discharge. Cats had significantly higher odds to achieve any ROSC [OR (95%CI) 2.72 (1.12–6.61), *p* = 0.028] and survive to hospital discharge than dogs [OR (95%CI) 4.87 (1.52–15.58), *p* = 0.008]. Patients had significantly lower odds of achieving any ROSC if CPA occurred during nighttime hours [OR (95%CI) nighttime = 0.52 (0.27–0.98), *p* = 0.043], and higher odds if CPA was witnessed [OR (95%CI) 3.45 (1.57–7.55), *p* = 0.002], if less people were involved in CPR efforts [OR (95%CI) 0.8 (0.66–0.96), *p* = 0.016], if pulses were palpable during CPR [OR (95%CI) 9.27 (4.16–20.63), *p* < 0.0005], and if an IV catheter was already in place at the time of CPA [OR (95%CI) 5.07 (2.12–12.07), *p* = 0.0003]. Odds for survival to hospital discharge were significantly higher if less people were involved in CPR efforts [OR (95%CI) 0.65 (0.46–0.91), *p* = 0.013] and for patients of the anesthesia service [OR (95%CI) 14.82 (3.91–56.17), *p* = 0.00007]. Overall, factors associated with improved CPR outcomes have remained similar since incorporation of RECOVER guidelines into daily practice. Witnessed CPA events and high-quality CPR interventions were associated with positive patient outcomes, emphasizing the importance of timely recognition and initiation of CPR efforts. An optimal CPR team size has yet to be determined.

## Introduction

There has been growing awareness of the importance of veterinary cardiopulmonary resuscitation (CPR) in recent years and the Reassessment Campaign on Veterinary Resuscitation (RECOVER) clinical guidelines on veterinary CPR were published in 2012. These formed the first evidence-based performance of CPR guidelines in dogs and cats, and the basis for more uniform CPR training amongst veterinary professionals (1).

Several factors associated with increased rates of return of spontaneous circulation (ROSC) and survival rates in veterinary CPR have previously been identified and include hospital, patient, and arrest variables such as the number of people involved in CPR efforts, arrest due to absolute or relative drug overdose, shorter time to CPR initiation, shorter CPR duration, palpable pulses during CPR, and presence of an intravenous catheter (IVC) at the time of cardiopulmonary arrest (CPA) (2–4). It is possible that with a more uniform approach to CPR in dogs and cats following implementation of the RECOVER guidelines, other factors that can influence patient outcome could be identified.

In order to study CPR objectively and compare patient population characteristics and patient outcomes across different centers, it is necessary to use uniform and standardized terminology. In human medicine, reporting guidelines for CPR event data were first published in 1991 as the “Utstein Style guidelines for uniform reporting of data from out-of-hospital cardiac arrest” (5). These guidelines are frequently updated, and several other Utstein-style guidelines for reporting of other specific CPR scenarios have been published since then (6–11). Most recently, Utstein-style guidelines on uniform reporting of in-hospital CPR in dogs and cats were released by the RECOVER initiative (12). To date, only one prospective veterinary study investigating a single monitoring variable as a predictor of improved CPR outcome employed veterinary Utstein-style definitions of ROSC (13).

The purpose of this prospective study was to re-evaluate previously described factors associated with a positive outcome in dogs and cats undergoing CPR at a university teaching hospital after implementation of the RECOVER veterinary CPR guidelines. A second aim was to identify novel factors associated with improved CPR outcome in dogs and cats as defined by the 2016 Utstein-style guidelines on uniform reporting of in-hospital cardiopulmonary resuscitation in dogs and cats (12).

## Materials and Methods

All dogs and cats that suffered from CPA and underwent CPR during the time period from December 2013 through June 2018 at the William R. Pritchard Veterinary Medical Teaching Hospital at the University of California, Davis, were prospectively enrolled in this study. CPA was identified if an animal was unconscious, lacked signs of both spontaneous circulation (lack of detectable pulse or heartbeat) and spontaneous breathing, or was showing occasional, gasping breaths only (12). CPR for the purpose of this study was defined as an attempt to restore spontaneous circulation by performing chest compressions and full reporting of case details is limited to animals receiving chest compressions (2, 12). If a patient suffered multiple arrests, only the first CPA and CPR events were described and analyzed for the present study. Patients were considered to have undergone CPA in the perianesthetic period if CPA occurred under general anesthesia, at induction of general anesthesia, at recovery (while waking up in the post-anesthesia recovery area until successfully extubated), as well as patients receiving procedural sedation. These patients were then subclassified as being cared for by the anesthesia service, or another service for analysis.

CPR at our institution during the study period was conducted in accordance with the RECOVER clinical guidelines for veterinary CPR with the exception of timing of vasopressor and atropine administration (1). In short, basic life support (BLS) was initiated immediately upon recognition of CPA and consisted of provision of circulation by chest compressions, orotracheal intubation, and positive pressure ventilation with 100% oxygen (1). Advanced life support (ALS) consisted of electrocardiogram (ECG) and carbon dioxide end tidal (EtCO_2_) monitoring, establishing vascular access, administration of epinephrine and atropine, and administration of reversal drugs or electrical defibrillation where indicated (1). Our institutional protocol is to administer vasopressors and atropine as soon as vascular access is available without waiting for ECG interpretation. Whenever possible, BLS and ALS would be performed simultaneously, but if numbers of rescuers were limited, ALS would be instituted only after BLS was underway.

Notes on important CPR events and timing were taken during the CPR efforts. Additionally, the supervising clinician filled out a purpose made data form (Supplementary Material) for each CPR event immediately upon completion of CPR efforts. The same data recording form was used for the entire time of the study. It was created in 2013 based on Utstein-style definitions and reporting templates for human CPR (14). Data recorded on the form included three sets of information surrounding the CPR event; animal, arrest, and outcome variables.

All animal, arrest, and outcome core variables, except for chest confirmation, were recorded as required by the veterinary Utstein-style guidelines (12). All supplemental animal and arrest variables were recorded as suggested by the veterinary Utstein-style guidelines (12). The supplemental outcome variables “date and time of extubation,” “30-day survival,” “date of death after discharge,” and “cause of death after discharge” were not recorded on the CPR data form. Additional values recorded that are not suggested by the veterinary Utstein-style guidelines included a suspected cause of arrest based on the supervising clinician's evaluation of the case at the time of CPA, the qualification of the supervising clinician and number of people involved in CPR efforts. The times from CPA to CPR in animals that arrived at the hospital dead on arrival (DOA) were estimated based on owner information and for patients with an unwitnessed in-hospital arrest were estimated based on the last time the animal was seen alive or vital parameters were recorded. Prognostic information is presented here while additional descriptive information on patient outcome can be found in an accompanying publication.

Missing data in any categories was retrospectively retrieved from the medical record. Some definitions on the CPR data form generated in 2013 that were derived from 2004 human Utstein-style guidelines and that did not exactly match the categories published in the 2016 veterinary Utstein-style guidelines were retrospectively adjusted prior to data analysis.

### Statistical Analysis

Patient outcomes of interest for multivariable logistic regression model selection were the achievement of any ROSC, sustained ROSC, and survival to hospital discharge. Thereby, any ROSC was defined as clinical signs of reinstituted effective circulation such as a palpable pulse, systolic blood pressure measurement >60 mmHg in the presence of a direct arterial blood pressure measurement waveform, or a marked increase in EtCO_2_ for the duration of at least 30 s (12). Sustained ROSC was defined as ROSC of ≥20 min duration (12). For each of the three outcomes, a potential effect on patient outcome was investigated for 20 independent variables of interest. Hospital variables of interest included time of the day during which CPA occurred (daytime 07:00 until 18:59 and nighttime 19:00 until 06:59), presence of a diplomate of the American College of Veterinary Emergency and Critical Care (DACVECC), American College of Veterinary Emergency and Critical Care (ACVECC) resident, or emergency room (ER) intern at the time of CPA, and maximum number of people involved in CPR efforts. Animal variables of interest included species, sex, weight, patient of the anesthesia service, and patient under general anesthesia at the time of CPA. Arrest variables analyzed included artificial airway, ECG monitoring, EtCO_2_ monitoring, invasive blood pressure monitoring, or any (peripheral or central venous) IVC in place at the time of CPA, in-hospital CPA, witnessed CPA, palpable pulses during CPR, time to rescue drug administration, and if open chest CPR was performed.

Five additional variables of interest could not be included in multivariable model selection due to the large number of missing values. These included time to CPR initiation, highest EtCO_2_ reading during CPR, time to intubation, whether the first detectable rhythm was shockable, and total duration of CPR.

Univariable analyses of all parameters were computed and ones with a significance of *p* < 0.10 were considered for further analysis. Multivariable logistic regression analysis was performed separately for any ROSC, sustained ROSC, and survival to hospital discharge using publicly available software (15). A forward and backward step-wise selection process was employed for variable selection, using Akaike information criterion followed by a manual search for more parsimonious models taking *p*-values into consideration. Odds ratios [OR; 95% confidence interval (CI)] were then computed. Results will be reported as OR (95% CI). *P*-values of < 0.05 were considered statistically significant.

## Results

### Patient Outcomes

**Figure 1:**
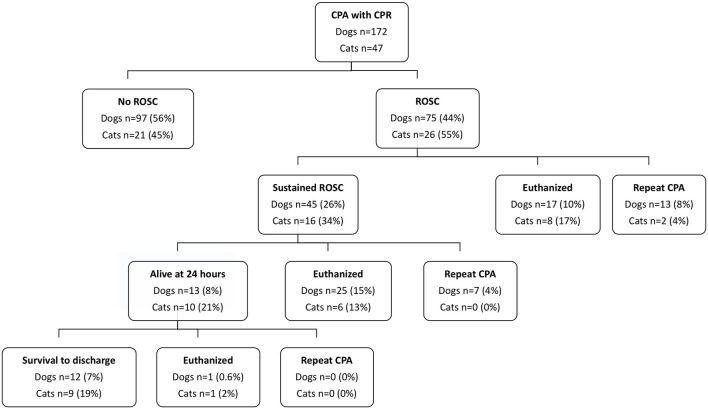
Outcome data for dogs and cats that suffered cardiopulmonary arrest and underwent cardiopulmonary resuscitation efforts. CPA, cardiopulmonary arrest; CPR, cardiopulmonary resuscitation; ROSC, return of spontaneous circulation.

Out of 177,125 canine and feline visits to the hospital during the study period, a total of 219 CPR episodes were recorded (0.12%) in 172 dogs and 47 cats. Of the patients that underwent CPR, 46% (101/219) achieved any ROSC and 54% (118/219) did not. Eighteen percent of patients (40/219) had ROSC ranging from 30 s to <20 min, 18% (40/219) had sustained ROSC for ≥20 min, and 10% (21/219) survived to hospital discharge; see Figure 1.

Of the animals that achieved ROSC for a duration of 30 s to <20 min, 63% (25/40) were euthanatized. Of the animals that achieved sustained ROSC, 34% (21/61) survived to hospital discharge. Of the animals that achieved sustained ROSC but that did not survive to hospital discharge, 54% (33/61) were euthanatized.

### Hospital Variables

Of the 219 patients included in this study, 62% (135/219) arrested between the hours of 7 a.m. and 7 p.m. and 38% (84/219) arrested between 7 p.m. and 7 a.m. For one CPR event, the training status of the supervising clinician was not specified. During the remaining CPR events the person with the highest level of training was a DACVECC in 58% of cases (126/218), an ACVECC resident in 20% (43/218) of cases, and an ER intern in 16% of cases (34/218). In the remaining 6% of cases, CPR was supervised by a non-ACVECC diplomate or resident. The median number of people involved in CPR efforts was 6 (*n* = 214, range 2 to 20).

### Animal Variables

Of the 219 patients suffering CPA, 172 were dogs and 47 cats. Ten percent of all animals included in this study were female intact (21/219), 33% were female spayed (72/219), 17% were male intact (38/219), and 40% were male castrated (88/219). Patient weight was estimated in 45% of all patients (98/219) either based on patient appearance, records of the referring veterinarian, or in-house medical records from a prior visit. Median patient weight in this study was 8 kg (*n* = 219, range 0.3 to 71 kg). Fifteen percent of dogs (25/172) and 28% of cats (13/47) were considered to have undergone CPA in the perianesthetic period. Of the dogs experiencing perianesthetic CPA, 64% (16/25) achieved any ROSC, and 36% (9/25) did not. Of the dogs that did not achieve ROSC after a perianesthetic CPA, the majority experienced CPA under general anesthesia (6/9, 67%), followed by CPA under procedural sedation (3/9, 33%). The majority of dogs that achieved ROSC underwent CPA while sedated for procedural interventions (6/16, 38%), 31% (5/16) under general anesthesia, 25% (4/16) during anesthetic recovery, and 6% (1/16) at anesthesia induction. Of the cats experiencing perianesthetic CPA, 77% (10/13) achieved any ROSC, and only 23% (3/13) did not. An equal number of cats that did not achieve ROSC experienced CPA under general anesthesia, during anesthesia recovery, or during procedural sedation (1/3, 33%). Of cats that achieved any ROSC, 40% (4/10) experienced CPA during procedural sedation, 40% (4/10) during general anesthesia, 10% (1/10) during anesthesia induction and 10% (1/10) during anesthesia recovery.

Prearrest severity of illness as assessed using the canine and feline Acute Patient Physiologic and Laboratory Evaluation (APPLE)_fast_ scores could only retrospectively be calculated in 33 of the 219 patients (15%), thus precluding inclusion of the APPLE_fast_ score in multivariable outcome models.

### Arrest Variables

At the time of CPA, a minority of patients already had BLS or monitoring equipment in place. An artificial airway was in place in 9% of patients (20/219), an ECG in 28% of patients (61/219), capnography or capnometry in 6% of patients (13/219), and invasive blood pressure monitoring in 5% of patients (11/219). Median time to intubation was recorded for 141 patients and was 1 min (range <1 min to 15 min). Sixty-eight percent of patients (149/219) had any intravenous catheter in place (peripheral venous catheter, central venous catheter, or both).

The majority of CPA events in this study population occurred in hospital (73%, 160/219) and 27% of patients suffered an out-of-hospital cardiopulmonary arrest (59/219). Seventy-five percent of all CPA events were witnessed (164/219), either by the owner or by a health care provider in hospital, and 25% of events (55/219) were unwitnessed. For three patients, information on rescue drug administration was missing from the records. Of the remaining 216 patients, 82% (177/216) received atropine and epinephrine and an additional 9% of patients (20/216) received epinephrine only and 4% of patients (9/216) received atropine only. In cases in which both drugs were administered, the median time to administration was the same for both medications (2 min, range 0 to 15 min for epinephrine and range 0 to 22 min for atropine). The median time to any rescue drug administration was 2 min (range 0 to 15 min).

Information on whether or not femoral pulses could be palpated during chest compressions was available for 183 patients. Of those, pulses could not be palpated in 48% of patients (88/183) but were palpable in 52% (95/183) of patients.

Open chest CPR was performed in 3% of patients (7/219) only whereas 97% of patients (212/219) underwent closed chest CPR. Of 192/219 CPR events, attending clinicians noted they believed open chest CPR was indicated in 20 cases (20/192, 10%), including the 7 cases in which it was performed. Reasons cited as indications for open chest CPR included patient size (*n* = 5), pleural space disease (*n* = 6), pericardial disease (*n* = 3), thoracic wall disease (*n* = 3), and ineffective closed chest CPR (*n* = 8). In the 13 cases, in which open chest CPR was not performed despite a perceived indication, reasons included owner wishes (*n* = 5), perceived futility by supervising clinician (*n* = 7), and generation of a palpable pulse with closed chest CPR despite large dog size (*n* = 1).

### Generation of Multivariable Models for Patient Outcomes

A total of five multivariable models were generated for the three patient outcomes of interest and results are summarized in Table 1. The arrest variable palpable pulse during chest compressions was strongly associated with patient outcome in univariate analysis (data not shown) but had 36 missing data points. Because of this strong association in univariate analysis, two models were created, one that included the variable presence of a palpable pulse (*n* = 177) and one that did not (*n* = 205) for the outcomes of any ROSC and sustained ROSC. For the outcome survival to discharge, given the low numbers of patients that achieved this outcome, only a model that did not include the variable presence of a palpable pulse could be created. Additionally, the arrest variables location of arrest and open chest CPR had to be removed from the model for survival to hospital discharge since zero patients that suffered an out-of-hospital cardiac arrest or that had open chest CPR performed survived to hospital discharge.

**Table 1:** Significant results of multivariable linear regression analyses for the three outcomes of interest, any return of spontaneous circulation (ROSC), sustained ROSC, and survival to hospital discharge in 172 dogs and 47 cats.

		**Pulses palpable included (*****n*** **=** **177)**			**Pulses palpable excluded (*****n*** **=** **205)**		
	**Variable**	**OR**	**95 % CI**	***p*-value**	**OR**	**95% CI**	***p*-value**
**ANY ROSC**							
Hospital variables	Night time arrest (7 p.m. to 7 a.m.)				0.52	0.27 to 0.98	0.043
	DACVECC present	2.29	1.01 to 5.18	0.046			
	Maximum number of people	0.80	0.66 to 0.96	0.016			
Animal variables	Feline	2.72	1.12 to 6.61	0.028			
	Weight (kg)				0.97	0.95 to 0.99	0.004
Arrest variables	Witnessed CPA				3.45	1.57 to 7.55	0.002
	IVC in place at time of CPA	5.07	2.12 to 12.07	<0.001	3.18	1.57 to 6.43	0.001
	Palpable pulses	9.27	4.16 to 20.63	<0.001			
**SUSTAINED ROSC**							
Hospital variables	Night time arrest (7 p.m. to 7 a.m.)				0.45	0.21 to 0.97	0.041
	Maximum number of people	0.79	0.64 to 0.97	0.023	0.79	0.66 to 0.94	0.008
Arrest variables	Witnessed CPA				3.46	1.27 to 9.46	0.016
	IVC in place at time of CPA	3.55	1.31 to 9.61	0.013			
	Palpable pulses	4.36	1.85 to 10.30	<0.001			
**SURVIVAL TO HOSPITAL DISCHARGE**							
Hospital variables	Maximum number of people				0.65	0.46 to 0.91	0.013
Animal variables	Feline				4.87	1.52 to 15.58	0.008
	Patient of the anesthesia service				14.82	3.91 to 56.17	<0.001

Two models were generated for any ROSC and sustained ROSC, one including the independent variable palpable pulses during CPR (n = 177) and one excluding the variable (n = 205). Only one model excluding the variable palpable pulses was generated for the dependent variable survival to hospital discharge. Odds ratios in the table are presented with 95% confidence intervals. P-values of ≤ 0.05 are considered statistically significant.

*CI, Confidence Interval; CPA, Cardiopulmonary Arrest; CPR, Cardiopulmonary Resuscitation; DACVECC, Diplomate of the American College of Veterinary Emergency and Critical Care; IVC, Intravenous Catheter; OR, Odds Ratio*.

### Variables Associated With Any ROSC

Three of the five *hospital variables* included in model selection were associated with patients achieving any ROSC. These included a negative association of increasing number of people involved in CPR efforts [OR 0.8 (0.66 to 0.96)], positive effect of DACVECC presence [OR 2.29 (1.01 to 5.18)] in the model including palpable pulses (*n* = 177) only, and negative association of night time CPA [OR 0.52 (0.27 to 0.98)] in the model excluding palpable pulses (*n* = 205). Of the five *animal variables* tested, cats were 2.72 times more likely to achieve any ROSC than dogs [OR 2.72 (1.12 to 6.61)]. Lighter patients were significantly more likely to achieve any ROSC, and every one-kilogram increase in body weight rendered ROSC 0.97 times less likely [OR 0.97 (0.95 to 0.99)]. The three *arrest variables* palpable pulses during CPR [OR 9.27 (4.16 to 20.63)], presence of an IVC at the time of CPA [OR 5.07 (2.12 to 12.07); OR 3.18 (1.57 to 6.43)] in both models, and witnessed arrest [OR 3.45 (1.57 to 7.55)] were significantly associated with a higher likelihood of achieving any ROSC.

### Variables Associated With Sustained ROSC

The two *hospital variables* nighttime CPA [OR 0.45 (0.21 to 0.97)], and number of people involved in CPR efforts were inversely correlated with sustained ROSC. Every additional person involved in CPR efforts lowered the odds of sustained ROSC by 0.79 [OR 0.79 (0.66 to 0.94)]. None of the *animal variables* tested were significantly associated with sustained ROSC. Of the *arrest variables*, having palpable pulses during CPR increased the odds of sustained ROSC in patients by 4.36 times compared to not having palpable pulses [OR 4.36 (1.85 to 10.30)]. Having an IVC in place at the time of arrest increased the odds of sustained ROSC by 3.55 times [OR 3.55 (1.31 to 9.61)], and patients were 3.46 times more likely to achieve sustained ROSC if their CPA was witnessed [OR 3.46 (1.27 to 9.46)].

### Variables Associated With Survival to Hospital Discharge

The *hospital variable* number of people involved in CPR efforts remained inversely associated with patient survival to hospital discharge, with every additional person being present making survival 0.65 times less likely [OR 0.65 (0.46 to 0.91)]. Of the *animal variables*, cats were 4.87 times more likely to survive to hospital discharge than dogs [OR 4.87 (1.52 to 15.58)], and patients of the anesthesia service were 14.82 times more likely to be discharged compared to patients of any other service [OR 14.82 (3.91 to 56.17)].

## Discussion

In this study, 46% of patients undergoing CPR achieved ROSC, which is comparable to previous veterinary studies reporting ROSC in 28–60% of dogs and 42–44% of cats, and to reports of ROSC rates of up to 53% for in-hospital cardiac arrest of human patients (3, 4, 16–18). Compared to previously published veterinary studies investigating factors associated with CPR patient outcomes, many factors could be confirmed in the present study (2–4). These include higher ROSC rates for patients with witnessed CPA, that had an IVC in place at the time of the arrest, and in which palpable pulses were generated during CPR (2). Higher odds of survival to hospital discharge were also found in patients that suffered a perianesthetic arrest, which has previously been reported (3, 4). In contrast to previous studies, in our study cats and patients with a lower body weight had increased odds to achieve any ROSC and cats were more likely to survive to hospital discharge. A higher number of people involved in CPR efforts has previously been found to be associated with better outcomes in cats, whereas in this study, we found decreased odds of ROSC and survival for both species if more people were involved in CPR efforts (4). Lastly, in the present study, suffering CPA during night time was associated with worse patient outcomes and the presence of a DACVECC during CPR efforts increased the odds for any ROSC. This is in contrast to a prior study conducted at our institution (2).

### Hospital Variables

In this study, decreased odds for the achievement of any ROSC and sustained ROSC were found in patients that suffered CPA between the hours of 7 pm and 7 am. This is in accordance with human studies demonstrating decreased rates of survival from adult and pediatric in-hospital cardiac arrest during nights and weekends (19–21). An effect of the time of CPA on patient outcome was demonstrated in our study even after controlling for variables such as medical education of the CPR leader, number of people present, interventions already in place at the time of arrest, witnessed arrest, and time to rescue drug administration that might be optimized during daytime. Variables that were not controlled for but have been hypothesized to explain poorer outcome of after-hours admissions in people include changes in after-hours staffing patterns and patient surveillance, impaired psychomotor skills of health care providers at night, as well as differences in BLS execution and it seems likely that these factors contribute to temporal differences in veterinary CPR outcomes as well (20, 21).

Our current finding is in contrast to a previous study conducted at our institution where patients had a greater rate of ROSC if they experienced CPA between the hours of 7 p.m. and 7 a.m., which at the time was attributed to more complex referred cases being presented to the hospital during daytime hours (2). Interestingly, in our current study, daytime CPA was only associated with greater odds of achieving ROSC and not with greater odds to survive to hospital discharge. Emergency room case load at our institution has increased since the initial study with more first opinion cases being presented during daytime and continuation of complex referral case admissions through the night. Additionally, doctor staffing has increased during the 7 p.m. to 7 a.m. hours with more experienced clinicians present compared to the previous study. It is possible that this contributes to a change in temporal outcome patterns and suggests that while CPR performance appears improved during daytime, survival to hospital discharge is more of a reflection of illness severity than of CPR performance. It would have been ideal to have a score of disease severity in a greater proportion of patients to compare the patient populations admitted to the hospital during daytime to that admitted at night and to that of previous veterinary studies.

When controlling for other variables including time of arrest, the presence of an ACVECC diplomate was associated with higher odds of achieving any ROSC in the statistical model including palpable pulses. In the human literature results are mixed as to whether a higher degree of medical training of the CPR team leader is associated with improved patient outcome (22–24). It has been suggested that any person assuming the role of team leader to streamline tasks and improve communication during a code is beneficial (25). In our study, neither the presence of an ACVECC resident nor an Emergency Medicine Intern was associated with increased odds of achieving ROSC. At our institution ACVECC diplomates are the most senior clinicians present in the emergency room or the intensive care unit on a given shift and it is plausible that more senior staff more naturally resumes a leading role during a CPR event, while this requires more courage from junior clinicians and veterinary technicians, leading to the absence of a clear CPR team leader. Lastly, the positive association of the presence of a DACVECC with ROSC was not maintained in all statistical models evaluated, as such the strength of this association is unclear.

Currently there is no consensus in human or veterinary medicine on optimal team size for CPR (26). The median number of people in the room during CPR efforts in this study was 6 and an increase in team size by one additional person led to a reduction in odds to achieve any ROSC or sustained ROSC of 20% and to a reduction in odds to survive to hospital discharge by 35%. It is likely that a minimum number of people is required to provide prompt and adequate CPR but in out-of-hospital CPA in people, it has been shown that patients resuscitated by teams consisting of three or more paramedics compared to two had reduced odds of survival to hospital discharge (27). Two high-fidelity manikin studies showed no significant differences in CPR performance between resuscitative teams of two, three, and four or between teams of three or six people, but the authors hypothesized that BLS performance might be negatively impacted by distractions caused by the performance of ALS procedures (25, 28). It is likely that more people will be present when animals have a longer CPR as over time more rescuers arrive to provide assistance. In addition, complex hospitalized patients with a higher illness severity often have multiple services involved in providing care. Consequently, these cases are likely to have more people participating in CPR efforts. In contrast, a previous veterinary study found improved ROSC rates for cats if an increasing number of people were involved in CPR efforts (4). This was an older study performed prior to the RECOVER guidelines which limits the ability for direct comparisons with the current study.

### Animal Variables

Cats had significantly greater odds of achieving any ROSC and almost five times greater odds of surviving to hospital discharge in certain models. While our model controlled for the fact that more cats than dogs were under general anesthesia at the time of CPA, it did not control for underlying disease severity and it is possible that the cats included in this study were less severely ill than dogs which could explain the increased odds of survival. Increased odds of any ROSC in cats could also be explained by a more compliant chest wall, making utilization of the cardiac pump mechanism for blood flow and improved circulation more likely. It would have been ideal to correlate these findings with scores for disease severity or EtCO_2_ measurements to validate these hypotheses. A significant association between patient weight and the achievement of any ROSC was also found. Every additional one kilogram of body weight made the odds of ROSC 3% less likely. This is in contrast to previous veterinary studies that were not able to demonstrate an association between patient weight and outcome (2, 3). It seems plausible that, similar to the feline species, thoracic wall compliance is higher in patients with lower body weight, making chest compressions more efficient and that this benefit could not previously be demonstrated due to more heterogeneous BLS execution prior to publication of the RECOVER guidelines (1).

Patients of the anesthesia service had almost 15 times greater odds to survive to hospital discharge compared to the other patient populations in this study. Of note, the category of perianesthetic arrest in this study was broad and included animals sedated for minimally invasive or diagnostic procedures. When animals that were under general anesthesia at the time of CPA were evaluated separately, they did not have improved ROSC or survival to hospital discharge rates. Patients suffering from CPA as a consequence of relative or absolute drug overdose including sedative or anesthetic drugs have previously been reported to be the patient population with the highest chance of achieving ROSC or to survive to hospital discharge and we were able to confirm higher odds of survival to hospital discharge in our study (2–4). It was previously hypothesized that patients suffering CPA in the perianesthetic period are less severely ill than other patient populations. Given that we found increased odds of favorable patient outcome even after controlling for variables such as monitoring devices already in place and therefore presumable shorter time to CPR initiation and BLS optimization, the results of the current study support that thought.

### Arrest Variables

Of the arrest variables, only the presence of an IVC was associated with increased odds of achieving any ROSC and sustained ROSC but not of survival to hospital discharge. This is in accordance with a previous veterinary study that showed that the presence of an IVC was statistically significantly associated with sustained ROSC in both dogs and cats (2). The reasons why the presence of an IVC at the time of CPA leads to better patient outcomes are difficult to determine. It has previously been hypothesized that IVC presence leads to earlier administration of rescue drugs or intravenous fluids, however, in the present study, presence of an IVC remained associated with better patient outcomes even after controlling for the variable time to administration of atropine or epinephrine, a variable that by itself was not associated with improved ROSC rates (2). In people, prompt administration of some antiarrhythmic drugs has been shown to be associated with improved ROSC rates but not rates of survival to hospital discharge, whereas high quality BLS, uninterrupted chest compressions, and early defibrillation have been shown to improve patient survival (29). Vascular access remains challenging to achieve during a state of decreased blood flow and it is possible that the presence of an IVC allowed rescuers to focus on more critical aspects of BLS and ALS instead of losing valuable time on attempts of IVC placement.

The majority of patients in this study had a witnessed CPA event and if CPA was witnessed, the odds to achieve any ROSC or sustained ROSC were ~3.5 times higher than in patients whose CPA was not witnessed. Suffering a witnessed CPA did not significantly change the odds of surviving to hospital discharge. Suffering CPA in-hospital (IHCA) compared to out-of-hospital (OHCA) did not change the odds of achieving any ROSC or sustained ROSC in the present study. Lastly, the effects of IHCA vs. OHCA on patient survival to hospital discharge could not be evaluated in a multivariable logistic regression model as zero patients with OHCA survived to discharge.

In people, outcomes of IHCA and OHCA vary considerably with OHCA survival more recently being reported to have improved to 10.4% compared to IHCA survival which reportedly ranges up to 34% (30–32). Part of this difference can likely be explained by a shorter time to CPR initiation if the patient is already at the hospital, a factor that has repeatedly been shown to positively affect patient survival in both IHCA and OHCA scenarios (33–36). A shorter time to CPR initiation is likely also the reason why patients with witnessed CPA events had higher odds to achieve ROSC in the present study. Interestingly, witnessed CPR events lead to higher odds of achieving ROSC but not higher survival to hospital discharge rates, which may reflect either the patients' underlying disease severity or owners wishes to euthanatize.

In contrast to human medicine, pre-hospital CPR by lay people or animal ambulance services is extremely uncommon in veterinary medicine and the vast majority of veterinary patients suffering OHCA will not be provided CPR until arrival to a veterinary clinic, even if the CPA event is witnessed by owners. The difference between IHCA and OHCA is therefore less of a measure of the quality of CPR provided in two different settings but more of a function of the time to initiation of CPR. Given the importance of rapid initiation of BLS measures for patient outcome, the veterinary IHCA population is similar to a patient population with a witnessed arrest as CPR can be provided sooner at a veterinary facility. The fact that no patients suffering OHCA survived to hospital discharge most likely is a reflection of patients with witnessed CPA events having higher odds of ROSC due to rapid CPR initiation as outlined above.

Patients with palpable pulses during CPR efforts had significantly higher odds of achieving any ROSC and sustained ROSC compared to patients in which palpable pulses were not generated during chest compressions. In early human guidelines for CPR, femoral pulse palpation was a commonly recommended practice to monitor efficacy of external chest compressions and it was assumed that palpable pulses indicate adequate antegrade arterial flow (37, 38). Since then, it has been discovered that during CPR, emptying of the right atrium in a retrograde fashion can occur, and the palpation of femoral pulses can signify retrograde venous blood flow rather than antegrade arterial flow as expected (38–40). The palpated pulses in our study population could therefore either represent adequate antegrade arterial flow or retrograde venous flow. However, if retrograde venous flow was present in a majority of cases, cardiac output would be expected to be severely compromised and a positive association with achievement of ROSC would seem unlikely. It therefore is more plausible that the positive association of pulse palpation with achievement of ROSC in both this study and a previous study from our institution indicate adequate forward blood flow in our patient population (2). EtCO_2_ monitoring is considered a more objective measure of the efficacy of chest compressions and it would have been ideal to correlate EtCO_2_ values and palpable pulse presence (13, 41). Even though an EtCO_2_ reading was obtained in 50% of patients in this study (109/219), the timing of EtCO_2_ acquisition and pulse palpation was unfortunately not standardized, making such a correlation impossible. Despite these limitations, our finding suggests that in the absence of a capnometer or capnograph for more objective monitoring, there might be a value in pulse palpation for the assessment of chest compression adequacy.

Time to rescue drug administration was not associated with increased odds of achieving ROSC or survival to hospital discharge in the present study in multivariate analysis. It is of note that only the time to epinephrine and atropine administration was included in the statistical analysis and therefore, no conclusions can be made regarding the value of early administration of reversal drugs, intravenous fluid administration, or other drug classes. Our finding supports the thought that BLS measures are of utmost importance for the achievement of ROSC and that provision of timely vasopressor support in the face of high quality BLS is secondary. However, the median time to rescue drug administration in our study was short at 2 min, reflecting our institutional protocol of early drug administration. Administration of rescue drugs at the 2 min mark after diagnosis of a non-shockable cardiac rhythm is currently recommended by the RECOVER guidelines (1). It is therefore possible that a potential detrimental effect of delayed drug administration could not be detected in our study and our finding should not be interpreted as evidence to delay rescue drug administration after diagnosis of a non-shockable rhythm.

## Limitations and Conclusions

Despite its prospective nature, this study has several limitations, most of which are inherent to studying CPR. A significant proportion of animals arrived at the hospital DOA (26%, 58/219) or suffered CPA shortly after arrival to the hospital. In these patients, limited physical examination or laboratory parameters were available, which in most cases precluded calculation of illness severity scores such as the APPLE_fast_ score so that unfortunately, severity of the underlying disease could not be controlled for in multivariable logistic regression models. Furthermore, several variables of interest with a potential effect on patient outcome, such as time to CPR, total time of CPR efforts, first documented arrest rhythm, or highest EtCO_2_ reading during CPR could not be included in the statistical models as initially intended due to large numbers of missing data points whereas for other variables some values were estimated by clinicians, such as the time from arrest to CPR initiation or patient weight. Both of these constraints further impact the assessment of factors associated with improved ROSC and survival to hospital discharge rates. While clinicians leading CPR efforts in this study were all familiar with the RECOVER guidelines, CPR execution was not standardized by protocol. Since clinicians filled out CPR data forms after conclusion of CPR efforts, it remains possible that information regarding the CPR event or patient outcomes was inaccurately recorded or that reported results were influenced by theoretical knowledge of the RECOVER guidelines. Lastly, euthanasia remains a factor confounding patient outcome and limiting already small case numbers further as it is unknown how many patients would have survived to hospital discharge after ROSC if post-CPA care had been provided to all.

Overall, at our institution, the factors associated with achievement of any and sustained ROSC and survival to hospital discharge were similar after incorporation of the RECOVER guidelines into daily practice compared to prior to their publication. Witnessed CPA events that occurred during daytime were associated with improved patient outcomes, highlighting the importance of decreasing the delay from CPA recognition to CPR initiation to improve CPR success rates. The importance of uninterrupted and high-quality BLS was furthermore suggested by patients doing better if pulses were palpable during chest compressions and if an IVC was already in place at the time of CPA. Lastly, patients experiencing CPA while under the care of the anesthesia service had significantly higher odds to achieve ROSC and survive to hospital discharge, which confirms that high-quality CPR should be provided to this patient group with high chances to survive a drug related CPA event.

## Data Availability Statement

The raw data generated for this study can be made available to any qualified researcher upon request to the corresponding author.

## Ethics Statement

Ethical review and approval was not required for the animal study because the study was of observational nature and included no interventions that would have changed the course of treatment for any animals in this study. CPR was performed based on verbal (in emergency situations) or written owner consent and CPR conduction was not altered by the observational study. Written informed consent for participation was not obtained from the owners because the study was purely observational in nature and did not interfere with standard of care in CPR provision at our institution. CPR was only performed if the owners wished for it and performance was based on verbal (in emergency situations) or written owner consent.

## Author Contributions

KH and SE designed the study, generated the CPR questionnaire, and critically revised the manuscript. SH compiled the data and wrote the manuscript. Statistical analysis was performed by a third party and results were interpreted by SH, KH, and SE. All authors read and approved the final manuscript.

### Conflict of Interest

The authors declare that the research was conducted in the absence of any commercial or financial relationships that could be construed as a potential conflict of interest.

## Abbreviations

ACVECC: American College of Veterinary Emergency and Critical Care
ALS: Advanced Life Support
APPLE: Acute Patient Physiologic and Laboratory Evaluation
BLS: Basic Life Support
CI: Confidence Interval
CPA: Cardiopulmonary Arrest
CPR: Cardiopulmonary Resuscitation
DACVECC: Diplomate of the American College of Veterinary Emergency and Critical Care
DOA: Dead on Arrival
ECG: Electrocardiogram
ER: Emergency Room
EtCO_2_: Carbon Dioxide end Tidal
IHCA: In-hospital Cardiac Arrest
IVC: Intravenous Catheter
OHCA: Out-of-hospital Cardiac Arrest
OR: Odds Ratio
RECOVER: Reassessment Campaign on Veterinary Resuscitation
ROSC: Return of Spontaneous Circulation.
