# Supplementary material for: Prospective Evaluation of Cardiopulmonary Resuscitation Performed in Dogs and Cats According to the RECOVER Guidelines. Part 1: Prognostic Factors According to Utstein-Style Reporting
Source: Front Vet Sci. 2019 Nov 7;6:384. doi: 10.3389/fvets.2019.00384 (PMC6854014; doi:10.3389/fvets.2019.00384)
Supplement: Supplementary file 1 [file Data_Sheet_1.PDF]

PLEASE NOTE: SAICU BLOOD GAS MEASUREMENTS CAN BE PERFORMED FOR NO CHARGE TO THE CLIENT DURING CPR

Cardiopulmonary Arrest Event Record

(To be completed at end of resuscitation by supervising clinician)

Patient Sticker

Date: _________ Weight _______kg
Estimated yes no

Time of arrest (please be exact) _________
If unknown please list as UNKNOWN
Enter vital signs taken in 4 hours pre-arrest if available

Time	Heart Rate	Blood Pressure*	Respiratory Rate	PaO2 (SpO2)	PaCO2 or PvCO2#	Temp

***Please note if MAP, SAP, DAP. #Please note if PaCO2 or PvCO2**

Pre-existing Conditions

☐ None
☐ Arrhythmia ____
☐ Cardiac Disease
☐ Hypovolemia
☐ Hypotension
☐ Hemorrhage
☐ Upper airway obstruction
☐ Pulmonary insufficiency
☐ Brain disease
☐ Spinal disease
☐ Peripheral neuro or neuromuscular disease
☐ Anemia
☐ Metabolic/electrolyte abnormality
☐ Kidney disease
☐ Hepatic disease
☐ Sepsis
☐ Other (please list)

Please Place in Folder Provided – Contact Kate Hopper if any questions

Interventions Already In Place At Time Of Arrest

☐ None
☐ Invasive airway
☐ Mechanical ventilation
☐ Pacemaker (Please circle – temporary or permanent)
☐ Chest tube(s)
☐ Anesthesia
☐ Procedural sedation
☐ Supplemental oxygen
☐ Other ____

Monitoring

☐ ECG
☐ Pulse oximetry
☐ ETCO2
☐ Direct blood pressure
☐ Indirect blood pressure

Vascular Access

☐ Peripheral venous
☐ Central venous
☐ Arterial
☐ Intraosseous

Continuous Infusion Drugs

☐ Dobutamine
☐ Norepinephrine
☐ Epinephrine
☐ Vasopressin
☐ Lidocaine
☐ Dopamine
☐ Norepinephrine
☐ Epinephrine
☐ Vasopressin
☐ Lidocaine

☐ Fentanyl
☐ Midazolam/diazepam
☐ Propofol
☐ Dexmedetomidine
☐ Other (list)
____
____
____
____

Patient Type

☐ Outpatient – not emergency
☐ Outpatient - emergency
☐ Hospital inpatient – wards
☐ Hospital inpatient – ICU

☐ Hospital inpatient – anesthesia service

Cardiopulmonary Arrest (CPA) Event – Immediate Cause

☐ Unknown
☐ Cardiac
☐ Hypovolemia
☐ Hypotension
☐ Hemorrhage
☐ Arrhythmia ______________________________________
☐ Anemia
☐ Respiratory insufficiency
☐ Upper respiratory tract obstruction
- ☐ Natural airway
- ☐ Artificial airway

☐ Neurologic disease
☐ Anesthesia/sedative drug related
☐ Metabolic/Electrolyte abnormality
- ☐ Acidosis pH < 7.1
- ☐ Glucose < 50 mg/dL
- ☐ Ionized Calcium < 1 mmol/L
- ☐ Sodium < 135 mEq/L
- ☐ Potassium > 6 mEq/L

☐ Toxin _______________________________
☐ [illegible]

In Hospital Arrest Yes No

Location of In Hospital CPA (specify where in the hospital CPA occurred)

____

Witnessed Arrest Yes No

How was CPA identified (check all that apply)

☐ No palpable pulse
☐ No palpable heart beat
☐ No auscultable heart beat
☐ No breathing evident (excluding agonal breaths)
☐ Non-responsive
☐ Acute collapse
☐ Acute drop in $ETCO_2$
☐ Loss of direct ABP waveform

☐ ECG changes

Was CPR (defined as chest compressions) performed? Yes No

If no – what was the reason?
☐ Owner wishes
☐ Considered futile
☐ Lack of equipment, people, etc
If no CPR performed – form is now completed – thank you

Time of initiation of CPR (please be exact): _______________________

Supervising clinician:
☐ ACVECC diplomate
☐ Other diplomate
☐ ACVECC resident
☐ Other resident
☐ ER intern

Approximate number of people ACTIVELY involved in CPR: ___________

Compression technique used for majority of CPR
☐ One hand around sternum
☐ Two hands 'circumferential technique
☐ Hands over the heart - lateral
☐ Hands over widest point of the chest – lateral
☐ Sternal compressions – dorsal
☐ Internal cardiac compressions

Average compression rate _______________________

Did compressions generate palpable pulses? Yes No

Was an impedence threshold device used? Yes No

End Tidal Carbon Dioxide

Was $ETCO_2$ monitored? Yes No Attempted but would not read

What was the highest value of $ETCO_2$ measured during CPR? ___________

Ventilation (check all that apply)

- ☐ Mouth to snout (note duration) ________________
- ☐ Mask (note duration) ________________
- ☐ ET Intubation
  - ☐ Already intubated
  - ☐ Time from beginning of CPR and successful intubation

Ventilation rate used ____________________

Equipment used for ventilation (check all that apply)

- ☐ Bain circuit
- ☐ Ambu bag
- ☐ Anesthesia machine

[illegible]

ECG: Time from beginning CPR to obtaining first document rhythm ________________

NOTE – only report first CPR event (from initiation of CPR to stop or ROSC > 2 mins)

First documented rhythm

- ☐ Asystole
- ☐ Pulseless electrical activity
- ☐ Pulseless ventricular tachycardia
- ☐ Ventricular fibrillation
- ☐ Other (specify) _______________________________

Details of changes in rhythm during CPR period

**Time from start of CPR**	**Rhythm**	**Was this change following defibrillation: Yes or No**

Defibrillation

Time from start of CPR	Rhythm Defibrillated	Defibrillation dose (J)	
		Monophasic	Biphasic

Notes
Any other details of this CPR you feel may be relevant.

Drug Administration

Epinephrine

- Time from beginning of CPR to first dose ___________
- Dose given __________
- Route of administration (IV, IO, IT, other) ____________
- Total number of doses ____________

Vasopressin

- Time from beginning of CPR to first dose ____________
- Dose given __________
- Route of administration (IV, IO, IT, other) ___________
- Total number of doses ____________

Atropine

- Time from beginning of CPR to first dose __________
- Dose given ___________
- Route of administration (IV, IO, IT, other) __________
- Total number of doses _________________

☐ Dextrose
☐ Insulin
☐ Lidocaine
☐ Calcium
☐ Sodium bicarbonate
☐ Naloxone
☐ Flumazenil
☐ Atipamezole
☐ Other (please specify)

Fluid Therapy

☐ None
☐ Hypertonic saline
☐ Isotonic crystalloid bolus
Total volume given _______________
☐ Synthetic colloid bolus
Type ___________________
Total volume given ______________
☐ Packed red blood cells
Volume given _____________
☐Plasma
Total volume given _____________

Open Chest CPR

Do you believe open chest CPR was indicated in this patient? Yes No

(For the first CPR event – if indicated later in pt management please indicate)

Indication for open chest CPR

☐ Size of patient
☐ Pleural space disease
☐ Pericardial effusion
☐ Thoracic wall trauma
☐ Ineffective closed chest CPR

Was open chest CPR performed? Yes No

If no - reason open chest CPR was not performed

☐ Owner wishes
☐ Considered futile
☐ Logistics (didn't have surgical pack available, etc)
☐ Other (please specify)

If Yes:

How long after starting CPR was open chest CPR initiated? ____________

Did internal chest compressions generate pulses? Yes No

What was the duration of open chest CPR? ______________

Did open chest CPR result in ROSC? Yes No

Were analgesics/sedatives needed? Yes No

Were any blood gases performed during CPR or within 10 minutes of ROSC?

Yes No

(Please attach any results and be sure exact time of blood collection, site of blood collection in relation to CPR/ROSC is noted)

CPR OUTCOME

Duration of CPR (from initiation to stop chest compressions). Please be exact. ________
Only report first 'CPR event' defined to end when stop efforts or ROSC > 2 mins (but do not include the 2 min of ROSC as part of the duration of CPR time recorded here)

Stopped CPR - No ROSC ☐

Reason to stop CPR (check all that apply)

☐ Owner request
☐ Futility due to underlying disease

☐ Futility due to lack of response of CPR
☐ Other ____

ROSC > 2 minutes? Yes No

Duration of ROSC (be exact) __________________

Note: ROSC ≤ 2 minutes before re-arrest is considered part of the same CPR event.

How many times did ROSC < 2 minutes occur during this CPR event? __________

Outcome of CPR event:
☐ Re-arrest
☐ Euthanasia
☐ Survival

Time of re-arrest or euthanasia: ___________________

Major reason for Euthanasia or stopping cpr efforts during a later arrest event:
☐ Poor prognosis
☐ Financial concerns
☐ Poor prognosis and financial concerns
☐ Unknown
☐ Other ______________________________

Please only complete one data form for the first CPR event a patient has in a 24-hour period. A CPR event is defined as CPR that ends in death or ROSC of > 2 minutes and occurs > 24 hours after any previous CPR event.

Post Successful ROSC Care

Was mechanical ventilation performed? Yes No
Reason for ventilation:

- ☐ Hypoventilation
- ☐ Hypoxemia
- ☐ Control of PCO_2, minimize VO_2, etc in compromised patient

Were anesthetic drugs needed to maintain intubation? Yes No

In 24 hours following ROSC:
Lowest temperature ____________
Highest temperature ____________

Lowest blood glucose ___________
Highest blood glucose ___________

Any arrhythmias evident in 24 hrs following ROSC? Yes No
Please list:

Complications (check all that apply)

- ☐ Difficulty in intubation
- ☐ ET tube displaced
- ☐ Excess airway fluid
- ☐ Loss of vascular access
- ☐ Loss of ECG leads
- ☐ Defibrillation issues
  - ☐ Machine malfunction
  - ☐ Failure to deliver shock (poor contact, inadequate dose etc)
  - ☐ Safety issues
- ☐ Incorrect drug dosing
- ☐ Other (please list)

* Please provide as many details are possible for complications – below

Was there a debriefing discussion following this event?

- ☐ Yes
- ☐ Sort of/partial
- ☐ No

COMMENTS / MORE INFORMATION:
Please make as many comments or add as many details about this CPA event as you can. Thanks!
